# Comparative analysis of 3D CT values for different lumbar pedicle screw trajectories

**DOI:** 10.1186/s13018-025-06046-x

**Published:** 2025-07-09

**Authors:** Yuanpeng Yue, Ce Dong, Anhang Zhang, Zhenyu Wang

**Affiliations:** Department of Orthopedics, Spine Surgery, The First Hospital of Jilin University, Jilin University, 1 Xinmin Street, Changchun City, 130000 China

**Keywords:** Traditional trajectory pedicle screw, Pedicle screw, Cortical bone trajectory screw, Transarticular surface screw, CT values, Three-dimensional trajectory

## Abstract

**Study objectives:**

The average CT values of the three-dimensional (3D) trajectories of transarticular surface (TAS) screws, traditional trajectory (TT) pedicle screws, and cortical bone trajectory (CBT) screws in the L4 and L5 vertebral bodies were evaluated, providing a radiological theoretical basis for the utilization of the transarticular surface screw trajectory (TAST) at these vertebral levels.

**Methods:**

A retrospective analysis was conducted on 3D CT images of the lumbar spine from 300 patients aged 21–70 years obtained from January to August 2024. Using Mimics 21.0 software, cylindrical models were constructed to simulate three types of pedicle screws, and the average CT values of the target cylindrical area (TCA) were measured. Further exploration of the relationships between factors such as age, sex, lumbar segment, and average CT values for the TCA was conducted.

**Results:**

Among the different age groups, the average CT value of the CBT group was the highest, followed by those of the TAS and TT screw groups (*P* < 0.05). Among patients of the same gender and age group, no significant difference was found in the average CT values of the same trajectory between the L4 and L5 vertebrae. Furthermore, in the 31–40 and 41–50 age groups, no significant difference was noted in the average CT values for the CBT between males and females. However, in the 21–30, 51–60, and 61–70 age groups, the average CT values of TT, CBT, and TAST were greater in males than in females (*p* < 0.05).

**Conclusions:**

The average CT values of the L4 and L5 vertebral bodies for the TAST exceed those for the TT but are lower than those for the CBT. This radiological study offers a theoretical basis for using TAS screws in the L4 and L5 vertebral bodies.

## Introduction

Dual-energy X-ray absorptiometry (DXA) is used for the assessment of bone mineral density (BMD); however, its accuracy may be compromised by spinal degeneration. This can result in an imprecise representation of the BMD of the vertebral body and the screw trajectory [1, 2]. The strong relationship between CT values and BMD enables CT values to offer a more precise assessment of BMD along the screw trajectory [3–5], as well as predict screw insertion torque and the risk of screw loosening [6–10]. Previous studies showed that the measured [3–5, 11] average CT values in regions of interest (ROI) of the TT and CBT were both based on two-dimensional planes. This approach failed to accurately capture the average CT values along the 3D trajectory of the screw. The use of TAS screws in the S1 vertebral body has been shown to be safe and feasible [12]. In patients who undergo laminectomy with Gill’s procedure and PLIF, the use of a CBT screw in combination with TAS screw (CBT-TASS) technology has minimally invasive advantages over TT pedicle screw placement via the Wiltse approach [13]. Notably, reports on the trajectory of lumbar TAS screws are limited. Therefore, this study utilizes 3D reconstruction techniques to accurately model the 3D trajectory of the pedicle screw, accurately assess the average CT values along these paths, and examine the BMD differences among three distinct trajectories in the L4 and L5 vertebrae from a radiological perspective. In addition, surgeons should be assisted in selecting a more suitable internal fixation method for patients to lower the risk of screw loosening and the extent of surgical injury. From a radiological perspective, these findings offer a theoretical basis for using TAS screws in the L4 and L5 vertebral bodies.

## Methods

### Obtaining 3D reconstruction data

This study involved a total of 300 patients between the ages of 21 and 70 years who underwent lumbar 3D CT scans at the First Hospital of Jilin University from January 2024 to August 2024. The study participants were categorized into five age groups: 21–30, 31–40, 41–50, 51–60, and 61–70 years. For each age group, 30 male and 30 female patients were randomly selected. Randomization was stratified by age and gender using a computer-generated sequence. Patients aged between 21 and 70 years had normal lumbar structure and symmetrical anatomical structure. Patients with spinal deformities, tumors, infections, trauma, a history of previous lumbar surgery, or a vertebral pedicle unsuitable for simulated screws, as well as those with other systemic diseases that may affect bone density, were excluded.

### Screw placement simulation

**Fig. 1:**
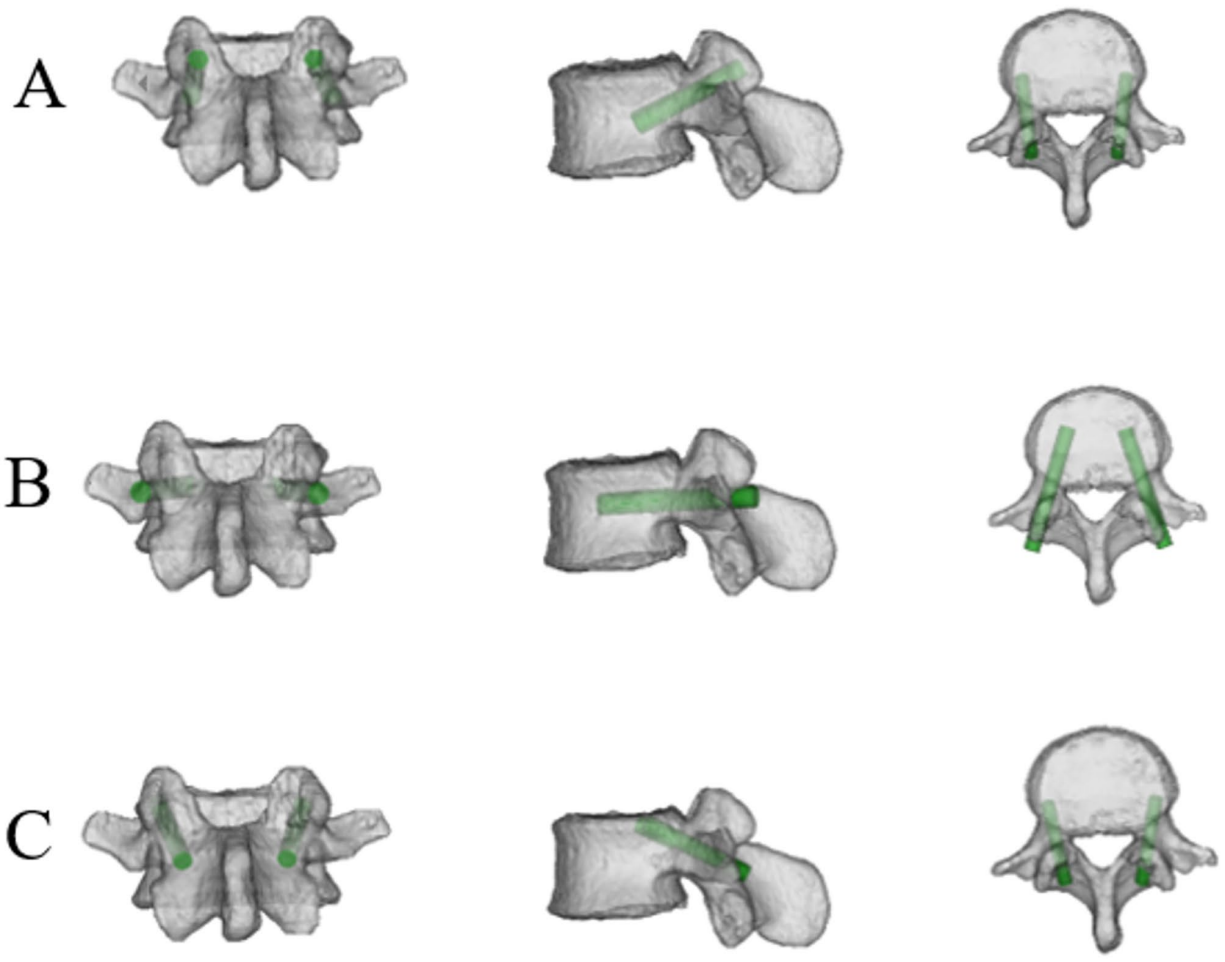
Model of the L4 vertebra and diagrams illustrating the trajectory from the front, sagittal and axial views. (**A**) TAS screws at L4; (**B**) TT pedicle screws at L4; (**C**) CBT screws at L4

**Fig. 2:**
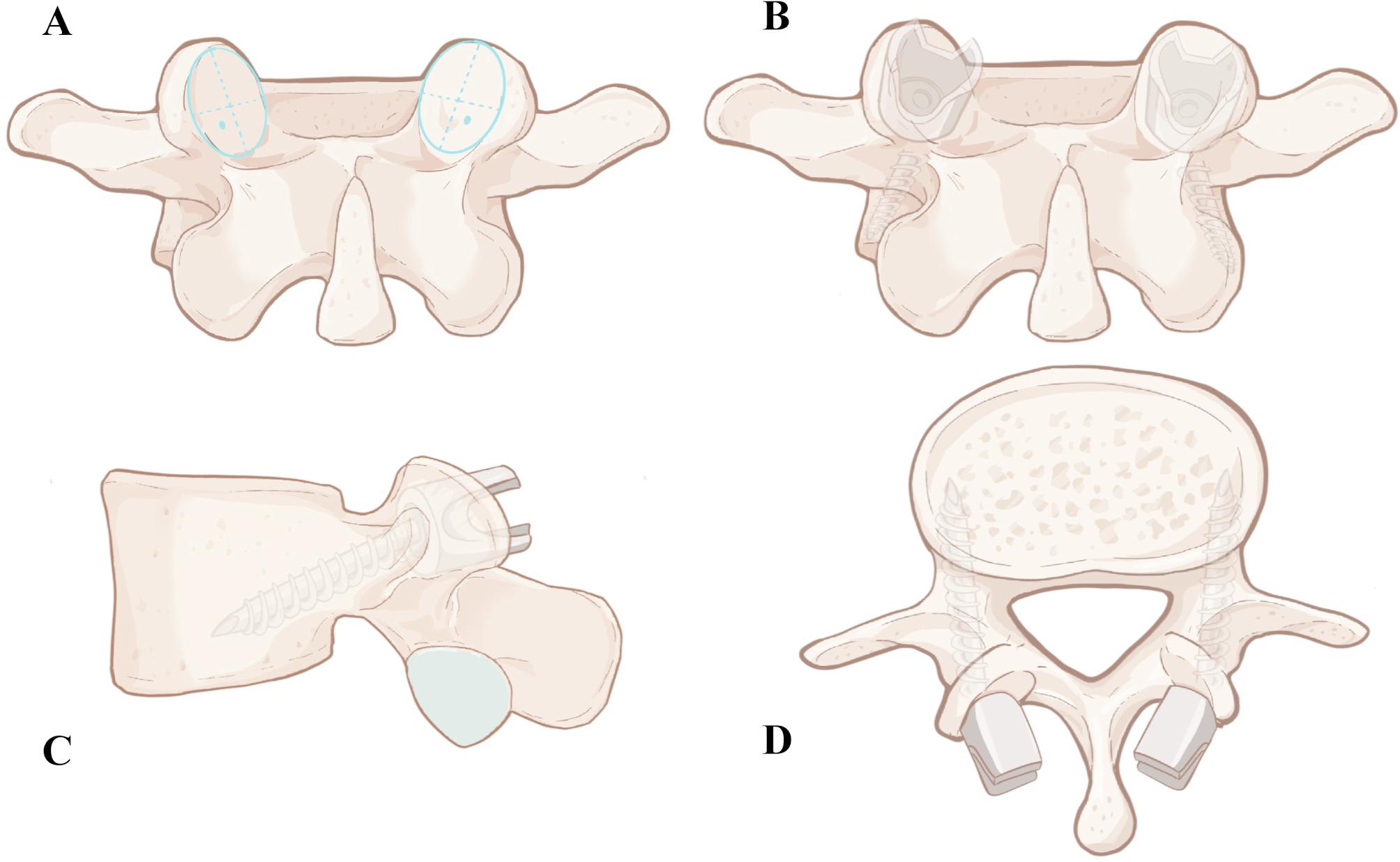
(**A**) The entry point for inserting the TAS screw into the vertebral body is located at the center of the lower outer quadrant of the articular surface of the superior articular process. (**B**) Front view of the TAS trajectory; (**C**) Sagittal view of the TAS trajectory; (**D**) Axial view of the TAS trajectory

We collected the imaging data via a Philips Brilliance 64 CT system for scanning, with a slice thickness and interval of 1 mm. Additionally, the DICOM data of patient lumbar 3D CT scans were imported into Mimics 21.0 software in this study. The “New Mask” function in the “Segment” module was subsequently selected, and the “Predefined Threshold Sets” were set to “Bone (CT)” to obtain the mask of the lumbar bone tissue. The “Split Mask " function was used on the mask of the lumbar bone tissue to segment out the 3D reconstructed L4 and L5 vertebrae. Next, the “Analyze” module was selected to create a cylinder simulating the pedicle screw, which was subsequently inserted into the L4 and L5 vertebrae in accordance with the screw trajectory. A cylindrical area along the screw trajectory was employed to perform CT measurements, and the bone properties of the bone-screw interface were shown to be sufficient in the cylindrical model [10]. This simulation model represents three different diameters and lengths of screws: a CBT screw (5.5 mm in diameter, 35 mm in length), a TT screw (6.5 mm in diameter, 40 mm in length), and a TAS screw (6.0 mm in diameter, 35 mm in length). Terai et al. observed that commonly used TAS screws in the S1 vertebra range from 6.5 to 7.0 mm in diameter and 35 to 40 mm in length [13]. Similarly, Akhgar et al. found that screw lengths between 32 and 35 mm are generally safe for preventing anterior wall perforation of the S1 vertebra [12]. Based on the entry point for the TAS screw in the S1 vertebral body [12, 13] and the multipoint cortical bone fixation of the CBT screw [14], the entry point for inserting the TAS screw into the vertebral body is located at the center of the lower outer quadrant of the articular surface of the superior articular process. The trajectory of the screw on the sagittal plane inclined caudally along the superior border of the pedicle, making contact with the lower wall of the pedicle and finally reaching the curvature of the vertebral body wall. (Figures 1A and 2). In terms of the traditional trajectory (TT) screw, the entry point was determined at the intersection of the horizontal midline between the lateral edge of the superior articular process and the transverse process, with the screw trajectory aligned parallel to the upper endplate of the vertebral body (Fig. 1B) [15]. The CBT screw entry point was confirmed at the junction of the center of the superior articular process and 1 mm below the inferior border of the transverse process [14, 16] (Fig. 1C).

### Measure the average CT values of the TCA

**Fig. 3:**
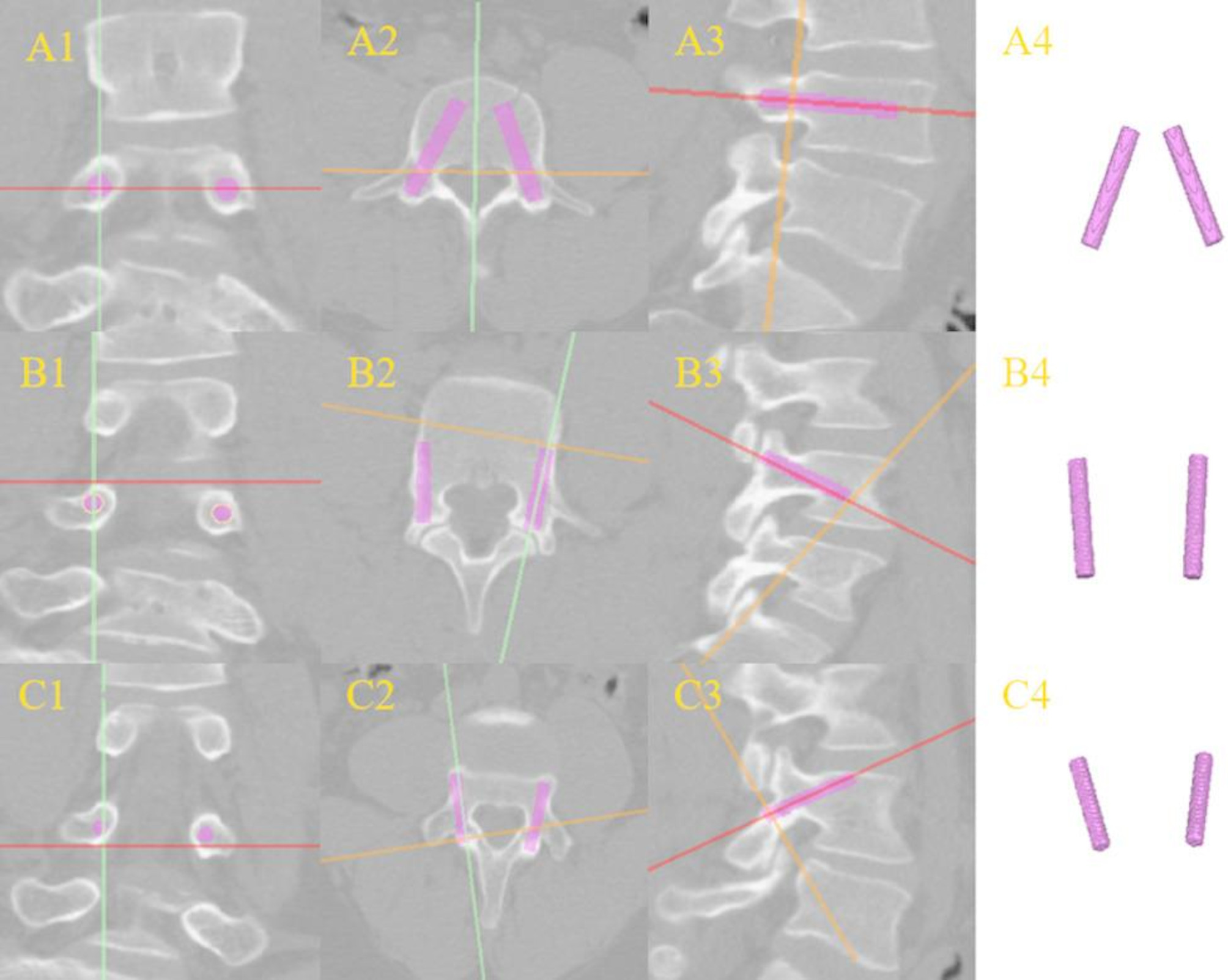
**A1**, **A2**, and **A3** represent the coronal, axial, and sagittal views of the TT, respectively, whereas **A4** shows the TCA of the TT extracted from the mask. **B1**, **B2**, and **B3** show the coronal, axial, and sagittal views of the TAS trajectory, respectively, and **B4** shows the TCA of the TAS trajectory extracted from the mask. **C1**, **C2**, and **C3** represent the coronal, axial, and sagittal views of the CBT, respectively, and **C4** represents the TCA of the CBT obtained from the mask

Cylinders were constructed to simulate three distinct types of pedicle screws, and the area where these simulated pedicle screws intersected with the bone tissue of the vertebrae was designated the target cylindrical area (TCA) (Fig. 3). The “object mask” function of the cylinder was used to isolate the TCA and generate the TCA mask, and the average CT value data of the TCA were obtained through the “properties” function of the TCA mask. Furthermore, all measurements in the study were carried out by a single spinal surgeon with expertise in lumbar anatomy and proficiency in the TT, CBT, and TAST techniques. All measurements were performed by a senior spine surgeon, with a one-week interval between the two independent measurements. The data were anonymized to avoid recall bias. The average CT values of the TCA were determined twice, and the final values were calculated by taking the average of the two measurements of the average CT values for the TCA. The intraclass correlation coefficient (ICC) was incorporated to strengthen the methodological reproducibility. A subsample of 34 patients (11.3%) was randomly selected from the 300 patients to determine the consistency of repeated measurements by the same physician. The sample size was determined using a formula (anticipated ICC = 0.85, confidence interval width ± 0.1).

### Statistical methods

SPSS 24.0 (IBM Corp, Armonk, NY, USA) was used to perform the statistical analysis. The Shapiro-Wilk test was employed in this study to assess the normal distribution of continuous variables. All continuous variables are presented as the means ± standard deviations (x ± s). To assess absolute consistency, the two-way mixed-effects model (ICC(3,1)) was chosen, with the single-measure ICC values and 95% confidence intervals (CI) reported. In addition, an ICC value greater than 0.75 was shown to indicate excellent consistency. Then, one-way ANOVA with Tukey’s HSD post hoc test was adopted for normally distributed data. The Kruskal-Wallis test with Dunn’s post hoc adjustment was used for non-normally distributed variables. *P* < 0.05 indicated statistical significance, with Bonferroni correction being used for multiple comparisons.

## Results

A two-way mixed-effects model (ICC(3,1)) was used to assess the consistency of repeated measurements by the same physician. The single-measure ICC values for TT, CBT, and TAST were 0.880 (95% CI: 0.766–0.941), 0.811 (0.643–0.905), and 0.859 (0.726–0.930), respectively, indicating excellent reproducibility of the measurements.

### Patient demographics

Detailed information regarding patient demographic characteristics is presented in Table 1.

**Table 1:** Demographic characteristics of all specimens

Age Group	Sex	Number	Mean age (years)	Mean height (m)	Mean weight (kg)
21–30	Male	30	24.27 ± 2.94	1.77 ± 0.60	75.33 ± 11.90
	Female	30	26.07 ± 3.18	1.65 ± 0.06	68.23 ± 12.64
31–40	Male	30	35.2 ± 1.90	1.73 ± 0.53	79 ± 15.44
	Female	30	35.47 ± 2.68	1.6 ± 0.05	60.2 ± 1.69
41–50	Male	30	43.37 ± 1.65	1.73 ± 0.04	79.8 ± 12.51
	Female	30	43.73 ± 1.76	1.61 ± 0.05	64.27 ± 4.55
51–60	Male	30	55.67 ± 2.61	1.71 ± 0.05	74.63 ± 7.95
	Female	30	54.2 ± 2.14	1.61 ± 0.04	61.93 ± 5.43
61–70	Male	30	65.67 ± 2.43	1.71 ± 0.06	72.13 ± 11.09
	Female	30	62.73 ± 2.42	1.61 ± 0.02	62.63 ± 5.81

### Comparison of average CT values of the TT, CBT, and TAST methods

**Fig. 4:**
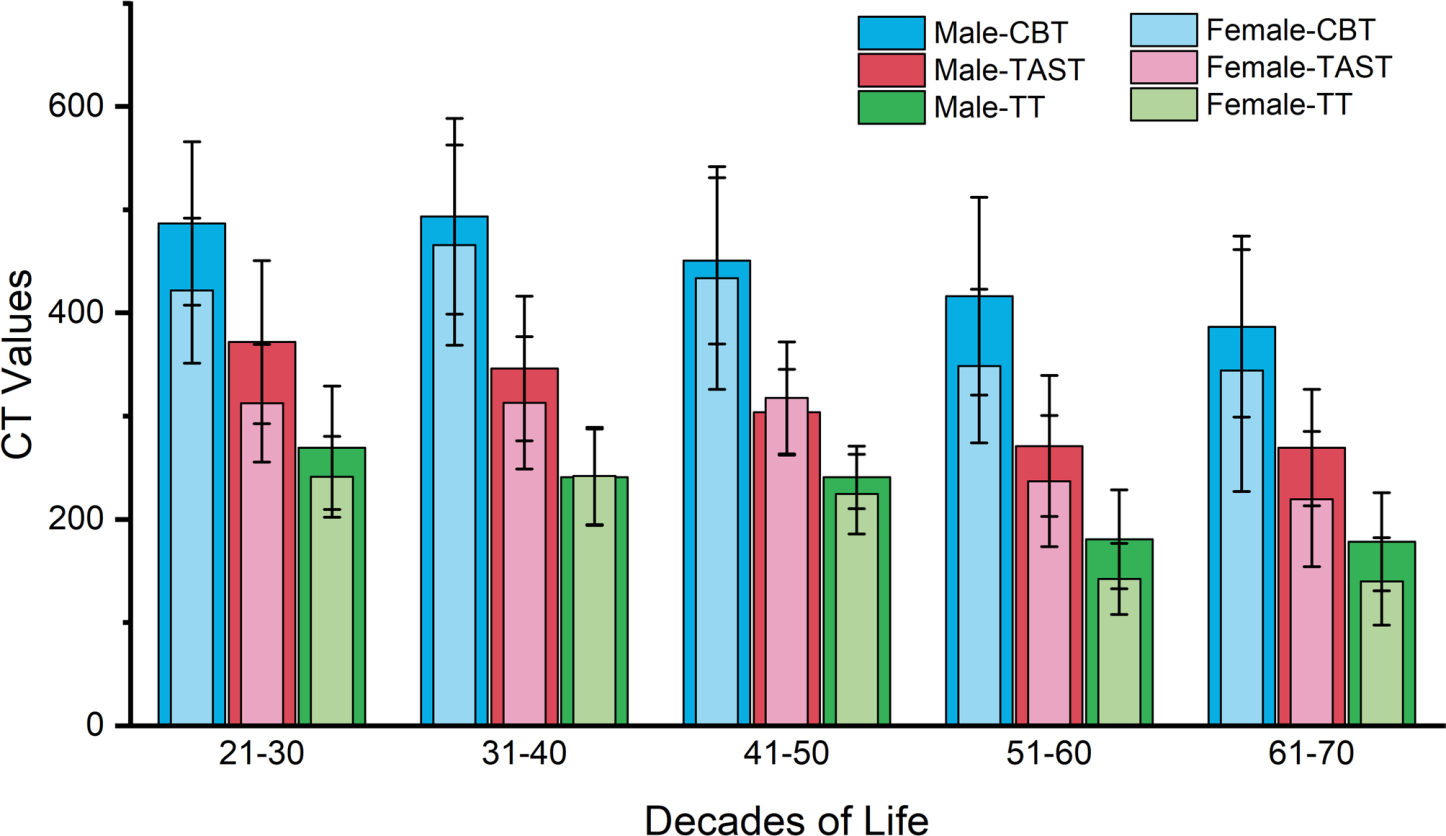
The average CT values of TT, CBT, and TAST in males and females across different age groups decreased as age increased

The average CT values for the L4 and L5 TCA, categorized by age group, sex, and screw trajectory, are displayed in Table 2; Fig. 4. The average CT values for the 3D trajectories in different age groups were ranked as follows: CBT > TAST > TT (*p* < 0.05). Among patients of identical gender and age groups, there were no significant differences between the same trajectories of L4 and L5 vertebral bodies (Table 3). Statistical analysis revealed no significant differences between male and female patients with CBT in the 31–40 and 41–50 years age groups (*p* > 0.05). In the 21–30, 51–60, and 61–70 years age groups, male patients presented higher TT, CBT, and TAST values than female patients (*p* < 0.05) (Table 4). As age increased, the average CT values of TT, CBT, and TAST decreased (Table 5).

**Table 2:** The average CT values of TT, CBT, and TAST were grouped based on age group, gender, screw trajectory, and the L4 and L5 vertebral bodies

Age Group	Sex	TT		CBT		TAST	
		L4	L5	L4	L5	L4	L5
21–30	Male	274.71 ± 59.28	264.27 ± 61.03	486.20 ± 100.44	487.77 ± 107.93	373.16 ± 77.61	370.73 ± 81.84
	Female	247.71 ± 43.67	235.83 ± 33.84	422.66 ± 71.64	420.96 ± 69.93	322.34 ± 54.37	303.22 ± 59.06
31–40	Male	249.30 ± 52.33	233.01 ± 39.65	514.72 ± 95.62	472.88 ± 90.58	359.88 ± 79.06	332.85 ± 58.28
	Female	252.48 ± 50.96	232.29 ± 41.38	478.28 ± 86.18	453.60 ± 107.05	328.85 ± 70.84	297.29 ± 53.22
41–50	Male	241.99 ± 40.25	239.75 ± 15.75	459.97 ± 88.81	441.41 ± 71.311	313.411 ± 54.11	294.58 ± 20.50
	Female	223.85 ± 40.82	225.70 ± 36.94	438.98 ± 119.41	428.78 ± 96.79	328.04 ± 60.43	307.77 ± 46.01
51–60	Male	180.04 ± 47.50	181.56 ± 49.25	421.83 ± 87.30	410.82 ± 104.87	271.79 ± 68.88	270.79 ± 69.10
	Female	145.54 ± 35.34	139.61 ± 33.84	363.76 ± 75.40	333.54 ± 71.28	246.62 ± 77.39	237.57 ± 45.2
61–70	Male	173.15 ± 39.83	184.06 ± 54.24	379.58 ± 75.48	394.25 ± 99.05	264.66 ± 51.80	274.67 ± 61.26
	Female	136.34 ± 38.71	143.93 ± 46.03	349.41 ± 107.42	339.41 ± 127.81	226.45 ± 65.33	213.12 ± 66.30
21–70	Male	231.00 ± 53.96	214.70 ± 48.35	453.62 ± 95.79	424.21 ± 93.14	321.71 ± 70.26	288.11 ± 56.03
		222.85 ± 51.80		438.91 ± 95.46		304.91 ± 65.63	
	Female	204.49 ± 69.60	190.71 ± 66.26	419.67 ± 111.99	402.27 ± 114.80	297.54 ± 85.90	278.19 ± 84.67
		197.59 ± 68.19		410.97 ± 113.55		287.87 ± 85.69	
Total		210.22 ± 61.80		424.94 ± 105.74		296.39 ± 76.74	

**Table 3:** Comparison of the average CT values of TT, CBT, and TAST in the L4 and L5 vertebral bodies

Age Group	Trajectory	L4	L5	t/Z	*p* value
21–30	TT	247.09 ± 43.68	235.83 ± 33.84	1.116	0.269
	CBT	422.66 ± 71.64	420.96 ± 69.93	0.093	0.926
	TAST	322.34 ± 54.36	303.22 ± 59.06	1.305	0.197
31–40	TT	252.48 ± 50.96	232.29 ± 41.37	1.684	0.098
	CBT	478.28 ± 86.18	453.60 ± 107.05	0.984	0.329
	TAST	328.85 ± 70.84	297.29 ± 53.22	1.951	0.056
41–50	TT	223.85 ± 40.82	225.70 ± 36.94	-0.183	0.855
	CBT	438.98 ± 119.41	428.78 ± 96.78	0.363	0.718
	TAST	328.04 ± 60.43	307.77 ± 46.02	0.257	0.149
51–60	TT	145.54 ± 35.34	139.61 ± 33.84	0.664	0.509
	CBT	363.76 ± 75.40	333.54 ± 71.28	1.595	0.116
	TAST	246.62 ± 77.39	227.57 ± 45.20	1.165	0.249
61–70	TT	136.34 ± 38.71	143.93 ± 46.03	-0.692	0.492
	CBT	349.41 ± 107.42	349.41 ± 107.42	0.328	0.744
	TAST	226.45 ± 65.33	213.12 ± 66.30	0.784	0.436

**Table 4:** Comparison of the average CT values of TT, CBT, and TAST between the male and female patients

Age Group	Trajectory	Male	Female	t/Z	*p* value
21–30	TT	269.49 ± 59.88	241.46 ± 39.15	-3.035	0.003
	CBT	486.99 ± 79.09	421.81 ± 70.19	-4.040	0.000
	TAST	371.94 ± 79.09	312.78 ± 57.10	-4.698	0.000
31–40	TT	241.15 ± 46.76	242.39 ± 47.13	0.144	0.886
	CBT	493.80 ± 94.72	465.94 ± 97.15	-1.590	0.114
	TAST	346.36 ± 70.19	313.07 ± 64.12	-2.713	0.008
41–50	TT	240.87 ± 30.33	224.78 ± 38.61	-2.539	0.012
	CBT	450.69 ± 80.40	433.88 ± 107.88	-0.968	0.335
	TAST	304.00 ± 41.66	317.90 ± 54.22	1.575	0.118
51–60	TT	180.80 ± 47.98	142.58 ± 34.43	-5.013	0.000
	CBT	416.32 ± 95.83	348.65 ± 74.32	-4.322	0.000
	TAST	271.29 ± 68.41	237.10 ± 63.57	-2.836	0.000
61–70	TT	178.60 ± 47.50	140.13 ± 42.34	-4.683	0.000
	CBT	386.92 ± 87.62	344.41 ± 117.16	-2.251	0.026
	TAST	269.66 ± 56.47	219.79 ± 65.60	-4.463	0.000

**Table 5:** Comparison of the average CT values of TT, CBT, and TAST among various age groups

Age Group	TT	CBT	TAST
21–30	255.825	447.015	340.880
31–40	239.040	480.045	331.040
41–50	231.315	430.985	302.815
51–60	148.340	373.115	236.870
61–70	147.335	361.775	230.670
H value	274.767	104.614	173.037
*p* value	0.000	0.000	0.000

*p*- value was calculated by Kruskal*-*Wallis test

### Specific values between CBT and TT (CBT/TT) and between TAST and TT (TAST/TT)

**Fig. 5:**
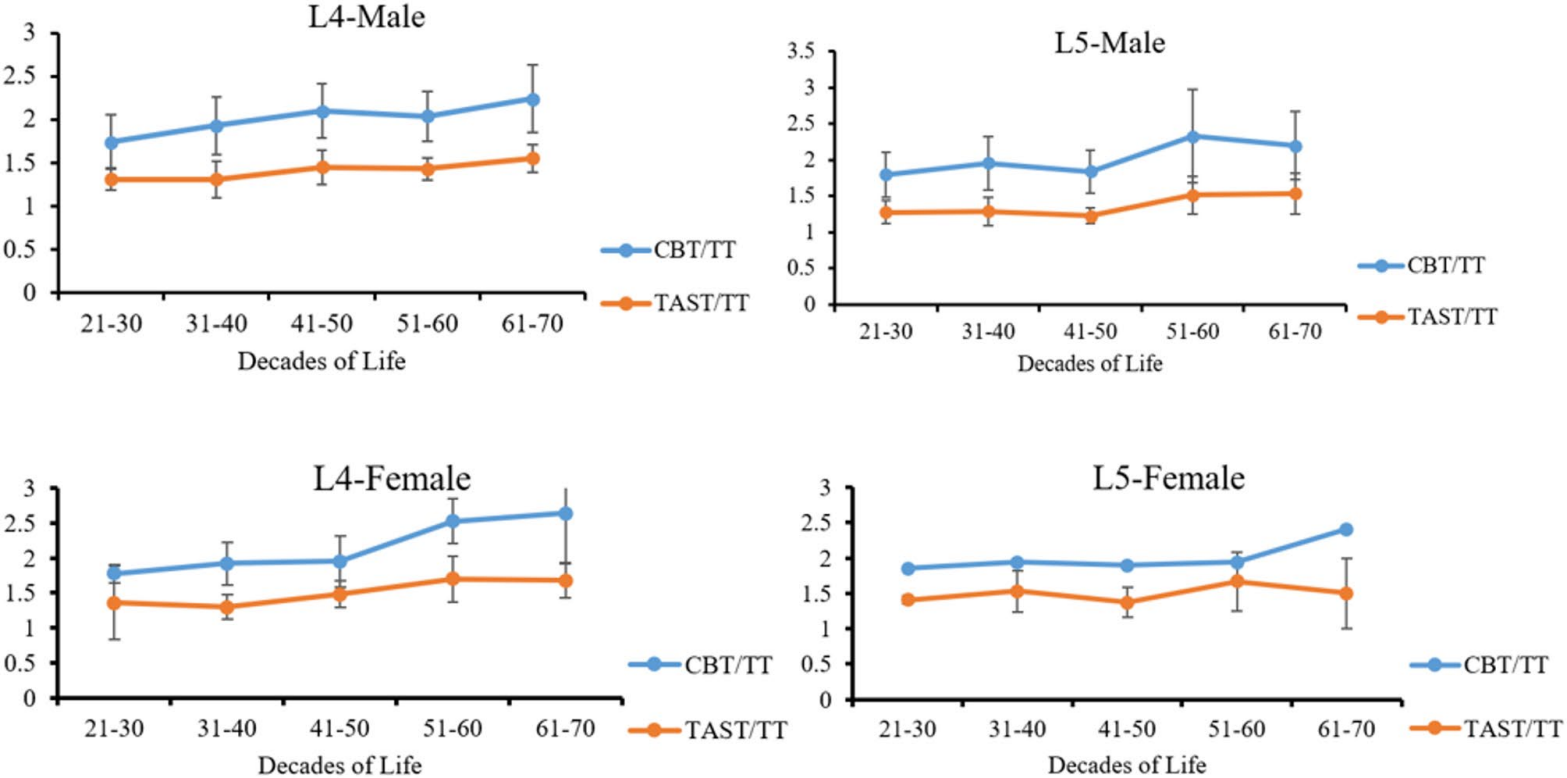
Variation in CBT/TT and TAS/TT within the L4 and L5 vertebrae of male and female patients

There were significant differences in CBT/TT, CBT/TAST, and TAST/TT among patients in different age groups (*p* < 0.05). Nevertheless, there were no significant differences in the CBT/TT or TAST/TT between the L4 and L5 vertebrae (*p* > 0.05). For individuals aged 51–60 years, the specific value between the TAST and TT was greater in female patients than in male patients (*p* < 0.05). Among the other age groups, no significant gender-based differences were found in CBT/TT or TAST/TT (*p* > 0.05) (Fig. 5).

## Discussion

### Comparison with the literature

For elderly osteoporotic patients requiring lumbar fusion and internal fixation surgery, poor bone quality might increase surgical risks, including screw loosening, refracture, and a reduced bone fusion rate [17]. Therefore, the preoperative analysis of vertebral bone mineral density (BMD) is important. Commonly, vertebral BMD is assessed via DXA, computed tomography (CT), quantitative CT (QCT), magnetic resonance imaging (MRI) [18], etc.; however, each method has advantages and limitations. The measurement of CT values of lumbar vertebral bodies is a simple and efficient technique for assessing bone quality. It has become one of the alternatives to DXA for evaluating lumbar BMD [6, 17, 19]. In addition, CT values are often used to assess the success rate of lumbar interbody fusion, cage subsidence, adjacent segment fractures, and pedicle screw loosening, as well as the risk of incidental durotomy [19]. During the perioperative period of lumbar fusion and fixation surgery, patients typically undergo lumbar 3D CT scanning. Preoperative 3D CT scanning provides more precise BMD information through the measurement of CT values and eliminates the additional costs and radiation exposure [18, 20]. Earlier studies measuring the average CT values of CBT, TT, and their fixation points often highlight the measurements of cancellous bone within the rectangular or elliptical regions of the vertebral body and pedicle [3–5, 11], which cannot accurately reflect the average CT values along the screw trajectory. Therefore, in this work, we employed 3D reconstruction technology to simulate the trajectories of three types of pedicle screws accurately and compared the differences in CT values among these three types of pedicle screw trajectories.

### Key findings

This radiological study quantified differences in bone mineral density distribution among three screw trajectory paths via 3D CT value assessment. Higher mean CT values suggest the potential for better bone-screw interface contact quality, which is often a significant predictor of favorable initial stability and insertion torque [6, 7, 10, 21–23]. However, the actual fixation strength of screws in vivo (e.g., resistance to pullout, loosening, and fatigue) and long-term stability constitute multifactorial issues [24]. It depends not only on local BMD but also on a combination of factors, including the screw design (diameter, length, thread morphology, and material), implantation technique, trajectory angulation, loading direction, biological integration at the bone-screw interface (osseointegration) [25], and overall spinal biomechanical environment (stiffness of the fused segment and motion of adjacent segments) [24, 26]. However, its actual biomechanical performance (e.g., pullout resistance and stability under physiological loading) at this level has not yet been validated, which is a crucial issue that must be addressed before its clinical adoption. Notably, although different CBT insertion techniques (e.g., variations in entry point and cephalad angulation) and screw sizes alter the proportions of contacted cortical and cancellous bone, leading to variations in mean CT values, the mean CT values of all CBT variants remained significantly greater than those of the TT trajectory [3–5, 11]. This fundamental difference stems from the CBT trajectory consistently traversing high-density cortical bone structures. The average CT value of the TAST is lower than that of the CBT, potentially because of the difference in the BMD of the isthmus and the superior articular process, as well as the uneven cortical bone mineral density of the lumbar pedicle wall and three-dimensional distribution of CT attenuation in the lumbar spine pedicle wall [27, 28]. The specific values between CBT and TT increase with age. Therefore, due to its high bone density and minimally invasive surgical advantages, CBT is more apt for adults than TT, especially in elderly patients. Compared with the cancellous bone in the facet joint, cortical bone BMD remains relatively stable with age, making it a reliable fixation structure [29]. Compared with that in men, the BMD of the facet joint in women is significantly affected by age (29). Degenerative changes, such as articular cartilage wear, subchondral bone sclerosis, and uneven bone density distribution due to degeneration [29–31], may negatively influence the initial fixation strength and long-term stability of screws near the entry point. Although the CT values measured in this study included the subchondral bone region, the structural heterogeneity of bone tissue resulting from degeneration may not be fully reflected by the average CT values. Future research should explore the optimization of TAS screw implantation techniques in degenerated facet joints (e.g., using navigation assistance and personalized planning) and establish more precise patient selection criteria based on imaging characteristics, such as facet joint degeneration grading and region-specific CT value thresholds [32].

### Clinical implications

The entry points of the TAS screws offer good visualization, enabling the screws to be inserted under direct observation of the nerve root. This improves the accuracy of screw placement within the vertebral arch [13]. Degeneration of lumbar facet joints may lead to a decrease in lumbar stability and limitations within the range of motion of the lumbar spine [33]. Hyperplasia and hypertrophy of the lumbar facet joints may cause stenosis of the lumbar intervertebral foramen and lumbar spinal canal, and the nerve roots passing through them may be compressed. Degeneration of facet joints increases the complexity and difficulty of surgery. In the process of lumbar surgery, if the facet joint is largely removed to reduce nerve compression, it will reduce the accuracy of positioning the insertion point of the TAS screw and increase the possibility of loosening the TAS screw. Degeneration and directional changes in the facet joint may lead to difficulties in identifying the entry point of TAS screws and misplacement of TAS screws [13]. Therefore, for patients with severe osteoporosis, elderly patients (especially female patients), patients with severe degeneration of the facet joints, and patients whose facet joints have undergone extensive resection during surgery, which may affect the stability of TAS screw fixation, the application of CBT screws may be more appropriate than the application of TAS screws.

In lumbar fusion and fixation surgery, TT pedicle screws are widely used because of their stable three-column fixation effect on the lumbar spine. In patients with osteoporosis, the loosening rate of TT pedicle screws is greater than that of CBT screws [34]. CBT screws are more suitable for minimally invasive surgery than TT pedicle screws, along with the advantage of a reduced risk of intraoperative and postoperative complications [35]. However, the learning curve for CBT is quite long, and the entry point for screws lacks easily distinguishable and repeatable anatomical landmarks [36], which may affect the accuracy and safety of screw placement during surgery. Compared with TT and CBT screw fixation, hybrid CBT and TT screw fixation has advantages in terms of minimal trauma and fixation strength in patients who have osteoporosis and degenerative spinal disease [37]. Moreover, the hybrid lumbar CBT and TT screw fixation technique can increase the biomechanical stability of the lumbar fusion segment and lower the risk of adjacent segment disease [38]. However, hybrid lumbar CBT and TT screws still fail to address the drawbacks of TT pedicle screw placement, which necessitates the exposure of a significant area of muscle tissue [39, 40]. Although midline lumbar interbody fusion (MIDLIF) is a minimally invasive surgical approach, the application of CBT screw fixation leads to the need to expose the surgical incision to the isthmus of the lower vertebral body to meet the requirements of CBT screw placement [41]. To further enhance the minimally invasive effect of MIDLIF, TAS screws are used to replace CBT screws for fixation of the caudal vertebra. CBT-TASS fixation technology can reduce the length of the surgical incision and the degree of invasiveness to muscle tissue, but whether it can achieve more minimally invasive surgical effects and favorable clinical effects needs further clinical research. Owing to the scarcity of relevant research on lumbar TAST supporting the adequate stability and biomechanical properties of the CBT-TASS fixation technique, further relevant research is strongly needed.

### Future directions and clinical translation

Using patient-specific preoperative CT data, individualized lumbar spine finite element models can be constructed by converting CT values along the 3D screw trajectory into bone material properties [42, 43]. These models simulate the pullout strength, as well as the stress and strain distributions within both the screw and the vertebra, for screws placed via three different trajectories under physiological loading conditions. The aim of this approach is to increase initial fixation stability by identifying the optimal “trajectory-screw” combination for a specific patient preoperatively, particularly in osteoporotic patients. The objective of such models is to predict the probability of screw loosening for a patient undergoing fixation via a specific screw trajectory, based on the preoperative CT values along the planned trajectory [44]. This approach facilitates the formulation of more proactive and individualized strategies for high-risk patients (e.g., those with severe osteoporosis or facet joint degeneration).

### Limitations

First, the study included only patients aged 21–70 years without spinal deformities or other diseases, which may limit the extrapolation of the results, especially in the context of elderly patients and complex diseases. Second, the study was based solely on 3D CT imaging, failing to reflect the influence of soft tissue status, ligament integrity, and other non-skeletal factors on screw stability, potentially leading to an incomplete assessment of the merits of screw trajectories. Third, while the radiological findings provide theoretical insights into the bone mineral density of lumbar TAS screw trajectories, the study lacks validation through biomechanical models or clinical environments. The biomechanical performance, long-term stability, and clinical outcomes of the use of TAS screws in lumbar fixation remain unverified, necessitating further experimental and observational studies. Fourth, the study was limited to a few commonly used screw sizes, not accounting for all clinically relevant specifications. Different screw specifications may exhibit varied behaviors depending on the trajectory. Furthermore, while the use of cylindrical regions to measure CT values simplifies the process, it deviates from the actual shape and thread structure of screws, which could influence the accuracy of assessing the true contact between screws and bone tissue. Future research could integrate multimodal imaging, examine additional screw specifications, and refine measurement models. Expanding the sample size and conducting prospective cohort studies and multicenter long-term follow-ups to evaluate clinical surgical outcomes and complications could provide more accurate guidance for clinical practice.

## Conclusions

To conclude, the average CT values of the L4 and L5 vertebral bodies for the TAST exceed those for the TT but are lower than those for the CBT. Moreover, this radiological study offers a theoretical foundation for the use of TAS screws in the L4 and L5 vertebral bodies.

## Data Availability

No datasets were generated or analysed during the current study.
